# Synergistic Effect of Quinic Acid Derived From *Syzygium cumini* and Undecanoic Acid Against *Candida* spp. Biofilm and Virulence

**DOI:** 10.3389/fmicb.2018.02835

**Published:** 2018-11-26

**Authors:** Subramanian Muthamil, Boopathi Balasubramaniam, Krishnaswamy Balamurugan, Shunmugiah Karutha Pandian

**Affiliations:** Department of Biotechnology, Alagappa University, Karaikudi, India

**Keywords:** *Candida* spp., synergism, biofilm, drug resistance, quinic acid, undecanoic acid, filamentation, ergosterol

## Abstract

In recent decades, fungal infections have incredibly increased with *Candida* genus as the major cause of morbidity and mortality in hospitalized and immunocompromised patients. Most of the *Candida* species are proficient in biofilm formation on implanted medical devices as well as human tissues. Biofilm related *Candida* infections are very difficult to treat using common antifungal agents owing to their increased drug resistance. To address these issues, the present study investigated the antibiofilm and antivirulent properties of *Syzygium cumini* derived quinic acid in combination with known antifungal compound undecanoic acid. Initially, antibiofilm potential of *S. cumini* leaf extract was assessed and the active principles were identified through gas chromatography and mass spectrometry analysis. Among the compounds identified, quinic acid was one of the major compounds. The interaction between quinic acid and undecanoic acid was found to be synergistic in the Fractional inhibitory concentration index (≤0.5). Results of *in vitro* assays and gene expression analysis suggested that the synergistic combinations of quinic acid and undecanoic acid significantly inhibited virulence traits of *Candida* spp. such as the biofilm formation, yeast-to-hyphal transition, extracellular polymeric substances production, filamentation, secreted hydrolases production and ergosterol biosynthesis. In addition, result of *in vivo* studies using *Caenorhabditis elegans* demonstrated the non-toxic nature of QA-UDA combination and antivirulence effect against *Candida* spp. For the first time, synergistic antivirulence ability of quinic acid and undecanoic acid was explored against *Candida* spp. Thus, results obtained from the present study suggest that combination of phytochemicals might be used an alternate therapeutic strategy for the prevention and treatment of biofilm associated *Candida* infection.

## Introduction

Most of the fungal infections in humans are caused by *Candida* species, particularly by *Candida albicans, C. tropicalis, C. glabrata*, and *C. parapsilosis* (Silva et al., 2017). In fact, these organisms are commensal of humans, but it may act as opportunistic pathogens under favorable circumstances. *Candida* infections are most frequent in low birth weight neonates, population with compromised immune system (AIDS, cancer, and diabetes patients) and transplant recipients. The infection levels are ranging from superficial skin infections to life threatening disseminated infections (d’Enfert et al., 2005; Nett et al., 2007). The major infection caused by *Candida* spp. is known as candidiasis, fourth most healthcare associated infections in world. Based on the site of infections, candidiasis is categorized into oral candidiasis in mouth, vulvovaginal candidiasis in vagina and invasive candidiasis affecting vital organs of the body. Among the 150 species of *Candida, C. albicans* is the major one responsible for the all types candidiasis (Pfaller et al., 2000). In the case of recurrent vulvovaginal candidosis, *C. albicans* (80–85%) and *C. glabrata* (10–20%) were most commonly isolated species (Sobel, 2007). Besides candidemia, oral thrush and denture stomatitis are other infections caused by *Candida* species (MacCallum, 2011). Furthermore, non-albicans *Candida* (NAC) species such as *C. tropicalis, C. glabrata C. parapsilosis*, and *C. krusei* are also observed as important nosocomial pathogens (Guinea, 2014). The prevalence of *Candida* spp. in denture wearers were found to be *C. albicans* (65%), *C. glabrata* (14%), *C. tropicalis* (11%), *C. parapsilosis* (7%), and *C. krusei* (2.7%) (Lyon et al., 2006). In some countries of the world, *C. tropicalis* has been regarded as second or third most causative agent of nosocomial candidemia in oncology patients with high mortality rate (Bizerra et al., 2008). Furthermore, *C. krusei* was most frequently found in leukaemic patients and bone marrow recipients, while *C. parapsilosis* is infrequent in cancer patients and capable of causing infection in children and urinary catheters (Krcmery and Barnes, 2002).

Most of the healthcare associated infections or hospital acquired infections are connected with biofilm formation on medical devices (Chandra et al., 2012). Biofilm is defined as structured communities of microorganism embedded within a self produced matrix consisting of extra polymeric substances (EPS) such as polysaccharides, lipids, proteins and extracellular DNA (Pereira de Mello et al., 2017). Fungi, in particular *Candida* spp. have been reported as third most pathogens in causing catheter associated infection which can form biofilm on both biotic and inert surfaces (Ramage et al., 2001; Deorukhkar and Saini, 2016). Indeed, *Candida* spp. are able to cause broad spectrum of biofilm mediated infection in implanted medical devices such as urinary catheters, dentures, prosthetic heart valves, contact lenses and silicone voice prostheses (Nett et al., 2015). In general, for the treatment of invasive candidiasis, amphotericin B-based preparations, azole groups and echinocandins are used. However, azoles alone mostly used for the treatment of mucosal infection caused by *Candida* spp. (Pappas et al., 2004). In the case of oral candidiasis, nystatin, amphotericin B, and fluconazole are the most frequently used drugs in antifungal therapy and occasionally ketoconazole and itraconazole are also used in the case of fluconazole resistant *Candida* strains (Garcia-Cuesta et al., 2014). Unlike planktonic organisms, cells encased in biofilm matrix exhibit 1000-fold increased resistance to antifungal agents (Rasmussen and Givskov, 2006). Azole groups such as fluconazole, ketoconazole, itraconazole, voriconazole, and posaconazole which target the ergosterol biosynthetic pathway are commonly used for antifungal treatments.3pc Shortly, fluconazole resistant *C. albicans, C. glabrata*, and *C. krusei* have been reported in immunocompromised patients (Cowen et al., 2000; Vandeputte et al., 2011; Terra et al., 2014). Similarly, antifungal resistance in NAC species against caspofungin and amphotericin B in the mixed species milieu as well as in transplant recipients has already been reported (Schwartz and Patterson, 2018; Vipulanandan et al., 2018). Hence, to overwhelm the current situation, alternative therapeutic agents superior to conventional antifungal agents, to reduce the antifungal resistance and virulence traits of *Candida* spp. are needed.

Historically, plants have been used as folk medicine to cure various diseases (Salini and Pandian, 2015; Banu et al., 2017). In recent past, compounds from natural resources and phytochemicals got more attention and have been shown as better anti-infective agents (Koh et al., 2013; Sivasankar et al., 2016). For example, 3-*O*-methyl ellagic acid from *Anethum graveolens* (Salini and Pandian, 2015), Undecanoic acid from *Hyptis suaveolens* (Salini et al., 2015), 2-Furaldehyde diethyl acetal from *Cocos nucifera* (Sethupathy et al., 2015), Vanillic acid from *Actinidia deliciosa* (Sethupathy et al., 2017a) and essential oils from *Cinnamomum tamala* (Banu et al., 2017), have been reported to comprise antimicrobial and antibiofilm properties against various human pathogens. In recent years, studies regarding antimicrobial peptides (AMPs) have been increasing tremendously because of their broad spectrum antimicrobial effect against virus, bacteria, fungi, protozoa and even cancer cells (Zhang and Gallo, 2016). AMPs are commonly found in both prokaryotes and eukaryotes as first line of defense. Unlike antibiotics, they target the cell membrane integrity and thereby affecting the protein, DNA and RNA synthesis Usually, AMPs are short peptides (5–100 aminoacids) proficient than antibiotics capable of controlling drug resistant pathogens, biofilm formation and persister cells in bacterial and fungal pathogens (Bahar and Ren, 2013; Mollica et al., 2018). At present, combined antimicrobial effect of phytochemicals and antibiotics have been studied against a variety of bacterial and fungal pathogens (Khan and Ahmad, 2012; Sivasankar et al., 2016). According to these reports, combination of phytochemicals decreased the dosage of drugs and modulates the drug resistance. Literally, fractional inhibitory concentration index (FICI) is used to determine the interaction between two drugs or phytocompounds using checkerboard method (de Castro et al., 2015). Quinic acid is a cyclic polyol, commonly extracted from different medicinal plants including *Eucalyptus globules, Hymenocrater calycinus, Tara spinosa, Ageratina adenophora, Urtica dioica*, coffee beans and barks of *Cinchona* genus (Gohari et al., 2010; Zhang et al., 2013). The compound has many advantages such as solubility in water, stability at room temperature, less cytotoxicity and also non-degradable nature in gut enzymes produced by bacteria (Rezende et al., 2015). Similarly, undecenoic acid, also known as undecylenic acid naturally occurs in human body (sweat), synthetically derived from castor oil. More than 50 years ago, the compound has been used for the treatment of skin infection caused by fungal pathogens (Anon, 2002) and approved by the Food and Drug Administration, United States for topical application. With this milieu, the present study aims to assess the antibiofilm potential of *S. cumini* and to evaluate *in vitro* synergistic effect of *S. cumini* derived quinic acid and undecanoic acid and their efficacy against *Candida* spp. biofilm and virulence.

## Materials and Methods

### Ethics Statement

This study was carried out in accordance with the recommendations of Ethical Guidelines for Biomedical research on Human Subjects, issued by Indian Council of Medical Research. The protocol was approved by the Institutional Ethics Committee, Alagappa University (Ref No: IEC/AU/2014/2). All participants gave written informed consent in accordance with the Declaration of Helsinki.

### Strains and Plant Collection

#### Fungal Strains and Drug Susceptibility Test

The details of reference strains and clinical isolates of *Candida* spp. used in the present study in the Table 1. The clinical isolates were collected from patients afflicted with *Candida* infection at the Government Hospital, Coimbatore, which includes urine (*n* = 4), vagina (*n* = 1), and sputum (*n* = 1) samples. The obtained cultures were isolated by *Candida* differential agar (Himedia, Mumbai) to check the purity of the cultures and identified at the species level by ITS sequencing. The GenBank accession numbers of CA1, CA3, CA4, CT1, CT2, and CT3 are MF423465, MF423466, MF423467, MF423462, MF423463, and MF423464, respectively. The reference strains and clinical isolates were maintained as glycerol stocks at -80°C. Also, all the strains were maintained on Sabouraud dextrose agar (SDA) (Himedia, Mumbai) plates at 4°C and prior to experiment, single isolated colonies of *Candida* spp. grown in yeast extract peptone dextrose (YEPD) broth at 37°C and 160 rpm for 24 h was used. For biofilm and filamentation assays spider broth (Mannitol 1%, K_2_HPO_4_ 0.2%, and Nutrient broth 1%) was used (Lu et al., 2011; Gulati et al., 2017).

**Table 1:** Antifungal susceptibility pattern of *Candida* spp. used in this study.

*Candida* strain	Minimum inhibitory concentration (MIC) μg mL^-1^				
	Fluconazole	Amphotericin B	Ketoconazole	Clotrimazole	Miconazole
*C. albicans* ATCC 90028^∗^	>100	2.5	100	50	25
*C. albicans* MTCC 186^∗^	>100 (512)	4	128	64	32
*C. glabrata* MTCC3019^∗^	>100	2.5	100	100	50
*C. tropicalis* MTCC 184	>100	5	100	100	50
*C. albicans* clinical isolate CA 1	64	4	32	32	16
*C. albicans* clinical isolate CA 2 MTCC 11802^∗^	>100	5	100	100	100
*C. albicans* clinical isolate CA3	128	8	16	16	16
*C. albicans* clinical isolate CA4	128	8	32	32	16
*C. tropicalis* clinical isolate CT 1	>100 (512)	32	128	64	64
*C. tropicalis* clinical isolate CT 2	>100 (512)	32	128	64	32
*C. tropicalis* clinical isolate CT 3	>100 (512)	32	64	128	32

*‘^∗^’ – Antifungal susceptibility of the strains has already been reported in our previous study (Muthamil et al., 2018)*.

The antifungal susceptibility pattern of all the *Candida* strains used in the study were assessed using broth dilution method described by Han et al., 2016 with slight modifications. Briefly, stock solutions of fluconazole (Himedia, Mumbai), miconazole nitrate, clotrimazole (MP Biomedicals, France), amphotericin B and ketoconazole (SRL, India) were prepared. In a sterile 96 well microtitre plate (MTP), antifungal agents at the concentration of 0.5–512 μg mL^-1^ were added in each well containing YEPD medium. All the 11 *Candida* strains (∼10^7^ cells) separately used as inoculum for each well and incubated at 37°C for 24 h. *Candida* strains without antifungal agents served as control and YEPD broth served as blank. After 24 h incubation, the cell density was measured at 600 nm using multifunctional spectrometer. The lowest concentration of the compound which showed maximum growth inhibition of pathogen was considered as MIC.

#### Plants Collection and Extract Preparation

*Syzygium cumini* plant leaves were collected from Karaikudi (10°5′54″ latitude/78°47′23″ longitude) and it has been identified as *S. cumini* by Dr. S. John Britto, The Rapinat Herbarium, St. Joseph’s College, Tiruchchirappalli, Tamil Nadu, India (Ref No: SM002). Primarily, the leaves were washed twice with distilled water and shade dried for 10–15 days. Ten gram of pulverized leaf powder was thoroughly extracted with 100 mL of methanol at room temperature for 24 h in orbital shaker (160 rpm). After 24 h, the filtrate was obtained by Whatman filtration and concentrated using rotary evaporator at 55°C. Further, 10 mg mL^-1^ of *S. cumini* methanolic extract (SCME) was prepared as a stock and used for further experiments (Muthamil and Pandian, 2016; Uysal et al., 2016).

### *S. cumini* Methanolic Extract (SCME): Extraction and Analysis

#### Determination of Biofilm Inhibitory Concentration (BIC) of SCME

Antibiofilm activity of SCME against the reference strain *C. albicans* (ATCC 90028) was assessed using the method by Subramenium et al. (2018) with slight changes. In brief, in a sterile 24 well polystyrene plate, ∼1 × 10^7^ cells from 24 h culture of *C. albicans* were used to inoculate 1 mL of spider broth containing varying concentration of SCME (25–500 μg mL^-1^) and incubated at 37°C for 24 h. *C. albicans* without SCME was considered as control and the spider medium alone served as blank. After incubation, the absorbance of culture was read at 600 nm to measure the changes in the cell density. Subsequently, loosely adherent planktonic cells and depleted media were removed and the biofilm cells were washed with sterile distilled water and air dried. Biofilm cells attached to the polystyrene surfaces were stained with 0.4% crystal violet aqueous solution for 5 min and the excess stain was removed by washing with distilled water. The crystal violet bound with biofilm was solubilized using 10% glacial acetic acid and its absorbance was measured using multifunctional spectrophotometer (Spectramax M3, Molecular Devices, United States) at 570 nm. The color intensity of crystal violet is directly related to the biofilm formation and decrease in OD indicates the biofilm inhibition (Subramenium et al., 2015). The percentage of biofilm inhibition was calculated using the formula:

% of inhibition = [(Control OD 570 nm - Treated OD 570 nm)/Control OD 570 nm] × 100.

#### Growth Curve Assay

To check the fungicidal effect of SCME on *C. albicans*, growth curve assay was performed (Banu et al., 2017). To a sterile 24 well polystyrene plate, 1 mL of YEPD broth supplemented with 1% *C. albicans* cells with and without SCME (at BIC) was added and incubated at 37°C. The absorbance was read spectrophotometrically at 600 nm at a regular interval of 3 h up to 24 h.

#### XTT Reduction Assay

In XTT reduction assay, 2,3-bis-(2-methoxy-4-nitro-5-sulfophenyl)-2H-tetrazolium-5-carboxanilide salt, along with menadione, was used to assess the effect of SCME on *C. albicans* metabolic viability (Sivasankar et al., 2016). Both SCME treated and untreated cells were washed twice with sterile PBS and resuspended in 100 μL of the same. Before experiment, XTT-Menadione solution was freshly prepared at the ratio of 12.5:1. Twenty five microliter of XTT-Menadione solution was added to SCME untreated and treated cell suspension and incubated in the room temperature for 5 h. After incubation, cells were separated by centrifugation and the absorbance of supernatant was measured at 490 nm. Sterile PBS along with XTT-Menadione solution served as blank.

#### Separation of Bioactive Fraction

The bioactive fractions were extracted by polarity based solvent extraction from non-polar to polar manner (n-hexane < benzene < petroleum ether < dichloromethane < chloroform < ethyl acetate < methanol) (Rajalaxmi et al., 2016). SCME was subjected to column chromatography using silica gel (60–120 mesh size, 50 cm × 2 cm) (Merck, United States). Subsequently, individual fractions were collected and used for activity based screening and the fraction which showed significant biofilm inhibition in *C. albicans* was used for further analysis.

#### GC-MS Analysis of Bioactive Fraction

The fraction with potential antibiofilm activity was analyzed by gas chromatography coupled with mass spectrometer (GC-MS) using AccuTOF Gcv equipment (SAIF, IITB, Mumbai, India). The GC separation was carried out in hp1 capillary column (length 30 m and diameter 0.25 μm), helium as carrier gas with a flow rate of 1 mL per minute. Temperature was maintained in the range of 100–280°C with ramp change of 5°C per minute. Compounds were identified by comparing with mass spectral database (NIST and WILEY Library, 2005).

### Effect of QA and UDA and Their Combination on Growth and Biofilm Formation

#### Determination of Minimum Inhibitory Concentration (MIC) of QA and UDA

Quinic acid and Undecanoic acid were obtained from TCI Chemicals (Chennai, India) and Sigma-Aldrich (St. Louis, MO, United States) respectively. Stock solutions of Quinic acid (QA) and Undecanoic acid (UDA) were prepared at the concentration of 10 mg mL^-1^ in methanol. The MIC of QA and UDA against *Candida* spp. was determined by broth dilution assay in 24 well microtitre plates (MTP) (Muthamil et al., 2018). Briefly, QA at the concentration of 12.5–800 μg mL^-1^ and UDA at the concentration of 2.5–160 μg mL^-1^ were added in each well containing YEPD medium. All the 11 *Candida* strains (∼10^7^ cells) separately used as inoculum for each well and incubated at 37°C for 24 h. *Candida* strains without compound(s) (QA or UDA) served as control. After incubation, the cell density (absorbance at 600 nm) was measured using multifunctional spectrometer.

#### Determination of BIC of QA and UDA

Standard crystal violet quantification method was used to assess the antibiofilm activity of QA and UDA against all the 11 *Candida* strains (Viszwapriya et al., 2016). In brief, QA at the concentration of 50–800 μg mL^-1^ and UDA at the concentration of 5–80 μg mL^-1^ were added in each well containing spider medium. *Candida* cells (∼10^7^ cells) were used as inoculum and incubated at 37°C for 24 h. Biofilm formation was quantified using crystal violet (0.4%) and percentage of biofilm inhibition was calculated as described earlier.

#### Checkerboard Assay

The combinatorial effect of QA and UDA against *Candida* spp. was studied by checker board method according to Dong et al. (2015) with minor changes. Briefly, in a sterile 96 well MTP, serial concentration of QA (25–800 μg mL^-1^) and UDA (1.25–80 μg mL^-1^) were added along with 200 μL of spider broth. Then 2 μL of inoculum was added to each well at the concentration of 1 × 10^7^ cells. A spider broth containing *Candida* cells without QA and UDA was maintained as control. FIC indices were calculated to evaluate the synergistic activity of the drug combinations (Matsumoto et al., 2014). FIC was calculated by dividing the BIC of combination of QA and UDA by the BIC of QA or UDA alone. The FICI was calculated by adding both FICs. Synergism and antagonism were defined by an FICI ≤ 0.5 and >4, respectively. Intermediate values such as 0.5–1.0 were considered as additive and 1.0–4.0 considered as indifferent (Odds, 2003; Matsumoto et al., 2014).

#### Cell Viability Assay

To assess the effect of QA-UDA combination against *Candida* spp. cell viability, XTT assay was performed (Kannappan et al., 2017). In this assay, equal amount of *Candida* cells were used to inoculate 1 mL of spider broth in the absence and presence of QA-UDA combination (at BIC) and incubated at 37°C for 24 h. After incubation, the cell viability of all the 11 *Candida* strains with and without QA-UDA combination was assessed by the method described above.

#### Microscopic Visualization of *Candida* spp. Biofilm

##### Light microscopy

*Candida* spp. biofilm was allowed to grow on 1 cm × 1 cm glass slide in 24 well MTPs in the absence and presence of QA-UDA combination (at BIC) for 24 h. After incubation, the slides were gently washed with distilled water and stained with 0.4% crystal violet for 5 min. The slides were again washed with distilled water and allowed to air dry. Then, the biofilm formed on the glass slides were observed under light microscope (Nikon Eclipse 80i, Japan) at 400× magnification.

##### Confocal laser scanning microscopy (CLSM)

Biofilm were allowed to grow on 1 cm × 1 cm glass slide in 24 well MTPs in the absence and presence of QA-UDA combination (at BIC) for 24 h. Then, loosely adhered cells were washed with sterile water and the biofilm cells on the surface of glass slides were stained with 0.1% acrydine orange (Himedia, Mumbai) for 5 min in the dark. The excess dye was removed by washing with distilled water. Three dimensional architecture of *Candida* spp. biofilm was visualized under CLSM (Carl Zeiss LSM710, Germany) and the additional factors such as biofilm biomass, average thickness and surface to volume ratio were determined using COMSTAT software (Gowrishankar et al., 2014).

### Effect of QA-UDA Combination on EPS Production

#### Extrapolymeric Substances Extraction

*Candida* spp. biofilm consists of self secreted Extrapolymeric substances (EPS) which act as protective barrier from antifungal agents. Thus, EPS was extracted from QA-UDA combination treated and untreated *Candida* strains by the method of Sivasankar et al. (2016) with minor changes. Briefly, *Candida* spp. was grown in the absence and presence of QA-UDA combination in YEPD broth (10 mL) at 37°C for 24 h. After incubation, the culture was centrifuged at 12000 rpm for 10 min to separate the cells and cell free culture supernatant (CFCS). Cell pellet was washed once with sterile PBS and suspended in 10 mL of isotonic buffer (10 mM Tris/HCl pH 8.0, 10 mM EDTA, 2.5% NaCl) and incubated overnight at 4°C. After incubation, the cell suspension was vortexed for 3 min and centrifuged at 12000 rpm for 10 min and supernatant was mixed with CFCS. Both Cell bound EPS and secreted EPS were precipitated by 3 volume of ice cold ethanol and incubated overnight at -20°C. Then, the EPS was collected by centrifugation at 12000 rpm for 10 min and the pelleted form of EPS was dried in rotary vacuum evaporator (Christ Alpha 2-4 LD plus, United Kingdom). The dried EPS was stored at 4°C for further quantification.

#### FTIR Analysis

Equal amount of dried EPS samples were taken from control and QA-UDA combination treated samples for FTIR analysis (Santhakumari et al., 2017). The samples were mixed with potassium bromide (KBr), 64 scans were taken from 4000 to 400 cm^-1^ with spectral resolution of 4 cm^-1^ in a FTIR spectrometer (Nicolet iS5, Thermo Scientific, Marietta, GA, United States). KBr pellet was used as a background reference.

#### Quantification of EPS Components

Extra polymeric substances from control and QA-UDA combination treated samples was dissolved in sterile distilled water and the amounts of polysaccharides, lipids, proteins and nucleic acids (extracellular DNA) were quantified using spectrometric methods (Padmavathi et al., 2015a). Total carbohydrates were quantified using phenol sulfuric acid method and the absorbance was taken at 490 nm. The proteins were estimated by Bradford method with bovine serum albumin as standard and optical density (OD) was read at 595 nm. Lipids were measured by phospho-vanillin method with cholesterol as a standard and the OD was taken at 545 nm. Extracellular DNA (eDNA) was quantified using nano spectrophotometer (Bio-Spec Nano, Japan) at 260/280 ratio with MilliQ water as blank. The effect of QA-UDA combination on *Candida* spp. EPS components were calculated using the formula:

% of inhibition = [(Control OD - Treated OD)/Control OD] × 100.

### Effect of QA-UDA Combination on Filamentation

The effect of QA-UDA combination on *Candida* spp. filamentous growth was evaluated using the method described by Muthamil and Pandian (2016). Briefly, spider agar supplemented with 1% fetal bovine serum (FBS) was used. Five microliter of 24 h grown *Candida* spp. with and without QA-UDA combination was inoculated on the agar surface and incubated at 37°C for 72 h. After incubation, the filamentous morphology of *Candida* spp. was photographed in gel documentation system (GelDoc XR+, Bio-Rad, United States).

### Effect of QA-UDA Combination Hydrolase and Ergosterol Production

#### Secreted Aspartyl Proteinases (SAPs)

Secreted aspartyl proteinases production in *Candida* spp. was qualitatively measured using bovine serum albumin (BSA) agar medium by the method of Inci et al. (2012) with slight changes. The medium consisted of 1% Glucose, 0.05% MgSO4, 2% agar, and 1% BSA and the final pH was adjusted to 4.5. Five microliter of 24 h grown *Candida* spp. in the absence and presence of QA-UDA combination was used to inoculate the agar surface and incubated at 37°C for 3–4 days. After incubation, SAPs production was determined by white opaque zone around the colonies and the diameter was measured by Hiantibiotic zone scale (Himedia, Mumbai). The plate images were taken in gel documentation system (GelDoc XR+, Bio-Rad, United States).

#### Phospholipase and Lipases

To determine the phospholipase activity of *Candida* spp. egg yolk agar method of Subramenium et al. (2018) was employed. Briefly, egg yolk agar medium was prepared in the composition of 1 L Potato dextrose agar, 10% NaCl, 0.005 M CaCl_2_ and 20% sterile egg yolk. Previously prepared *Candida* cell suspension with and without QA-UDA combination was used for inoculation and the plates were incubated at 37°C for 5 days. The presence of precipitation zone around colonies indicates the phospholipase activity.

Lipase production in *Candida* spp. was determined using tributyrin agar method described by Ramnath et al. (2017) with minor changes. The medium consisted of peptone 0.8%, yeast extract 0.4%, NaCl 0.3%, Agar 2.0% and after autoclaving tributyrin 0.2% was added at 55°C. Five microliter of QA-UDA combination treated and untreated *Candida* spp. culture was used to inoculate the center of agar surface and the plates were incubated at 37°C for 48 h. After incubation, zone of clearance around colonies was measured by Hiantibiotic zone scale. Additionally, CFCS were collected from *Candida* spp. grown in the absence and presence of QA-UDA combinations. Secreted extracellular lipases were quantified using p-nitrophenyl palmitate as substrate and the absorbance was measured at 410 nm (Sethupathy et al., 2017b).

#### Ergosterol Extraction and Quantification

Changes in ergosterol content of *Candida* spp. in the absence and presence of QA-UDA combination were quantified using UV Spectrophotometer (Shafreen et al., 2014). The yeast cell suspension (10^7^ cells mL^-1^) was used to inoculate YEPD (10 mL) supplemented with BIC of QA-UDA combination. The assay mixture was incubated at 37°C for 48 h with shaking condition. The cells at OD_600_ = 1.2 were harvested by centrifugation at 10,000 rpm for 5 min and washed once with distilled water, then dried and weighed. The cells were suspended with 3 mL of 25% alcoholic potassium hydroxide and vortexed for 1 min. The tubes were incubated at 80°C for 1 h and later were allowed to cool at room temperature. Then, the total sterol was extracted with 1 mL of distilled water and 3 mL of n-heptane. The mixture was vortexed constantly for 10 min until the distinct layer of n-heptane was observed. The clear layer of heptane was transferred to a clean borosilical tube. Further, the extracted sterol 20 μL was diluted up to fivefold using 100% ethanol and scanned spectrophotometrically between 200 and 300 nm with a UV spectrophotometer (UV 2450, Shimadzu, Japan). The sterol content in both control and treated cells was quantified using sulfuric acid-vanillin method described above.

### Effect of QA-UDA Combination on Virulence Genes Expression of *C. albicans*

#### Real Time PCR

To check the effect of synergistic combination of QA and UDA on *C. albicans* gene expression, RNA was isolated from control and QA-UDA combination treated cultures using hot phenol extraction method (Collart and Oliviero, 2001). Then, the RNA samples were reverse transcribed into cDNA by High capacity cDNA Reverse Transcription kit (Applied Biosystems, United States). Real time PCR was performed for candidate genes *als1, als3, cdr1, mdr1, erg11, flu1, nrg1, sap1, sap2, sap4, tup1, hwp1, eap1, efg1, cst20, ras1, ume6, hst7*, and *cph1*) involved in biofilm formation and virulence production in *C. albicans* (Supplementary Table S1) in 7500 Sequence Detection System (Applied Biosystems, United States). The primers were mixed separately with SYBR Green kit (Applied Biosystems, United States) at a predefined ratio and the PCR cycle had the temperature pattern of initial denaturation at 94°C for 10 min, denaturation at 94°C for 1 min, annealing at 55 and 60°C for 1 min and extension at 72°C. The expression of candidate genes were normalized against ITS gene (∼540 bp) expression which was taken as housekeeping gene and relative gene expression level was determined using the ΔΔ*C*T method.

### Effect of QA-UDA Combination on *C. elegans* Survival and *in vivo* Biofilm

#### *C. elegans* Survival Assay

The combinatorial effect of QA and UDA on *Candida* spp. virulence was assessed using a simple eukaryotic model organism *C. elegans*, which is frequently used for toxicology research (Leung et al., 2008; Viszwapriya et al., 2016; Hunt, 2017; Sharika et al., 2018). Three reference strains viz. *C. albicans* ATCC 90028, *C. tropicalis* MTCC 184, and *C. glabrata* MTCC 3019 were taken for *in vivo* studies. The nematodes at L4 stage were divided into five groups, with each group containing ∼10 worms suspended in 1 ml of M9 buffer. *Escherichia coli* OP50 (∼1 × 10^3^ cells) was used as a food source for the worms. The toxicity of QA-UDA combination was assessed using (i) Control group (*E. coli* OP50 alone), (ii) Vehicle control group (Methanol) and (iii) *E. coli* OP50 and QA+UDA (at BIC). Fourth and fifth groups were supplemented with *Candida* cells (∼1 × 10^7^ cells) in the absence and presence of QA-UDA combination, respectively. The survival rate of worms were monitored every 4 h using a stereo microscope (Nikon SZ-1000, Japan). The worm was considered to be dead when it failed to respond to external stimuli.

#### Microscopic Analysis of *C. elegans*

The combinatorial effect of QA and UDA on *Candida* spp. in *in vivo* biofilm formation and *C. elegans* physiology was evaluated by light microscopy (Gowrishankar et al., 2015). The worms were exposed to *Candida* strains in the absence and presence of QA-UDA combination at BIC for 12 h at 20°C. Then, the worms were washed thrice in M9 buffer and placed in glass slide with 0.01% sodium azide and visualized under light microscope (Nikon Eclipse 80i, Japan) at 400× magnification. Worms exposed to *E. coli* OP50 alone was kept as a control.

### Statistical Analysis

All the experiments were performed in triplicates and the data were presented as mean ± standard deviation. For all experiments, statistical differences between control and treated samples were analyzed with one way ANOVA followed by Dunnett’s test using Graphpad prism software 7.04. The *P*-value < 0.05 was set as statistically significant.

## Results

### Effect of SCME on the Biofilm and Growth of *C. albicans*

**FIGURE 1:**
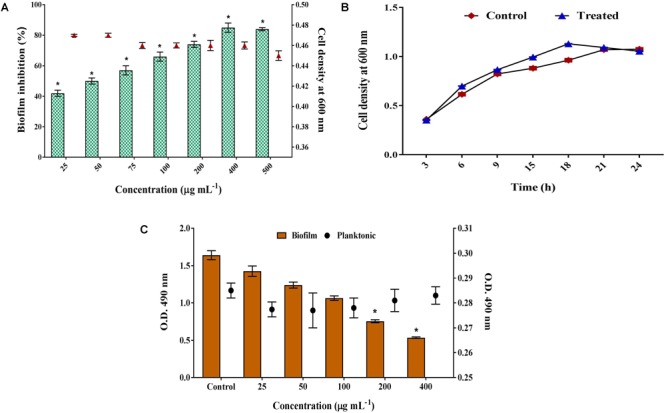
Inhibitory effect of SCME on *C. albicans* growth and biofilm. **(A)** Non-fungicidal antibiofilm effect of SCME at varying concentrations. The maximum biofilm inhibition observed at the concentration of 400 μg mL^-1^ was identified as BIC. **(B)** Growth curve of *C. albicans* planktonic cells in the absence and presence of SCME after 24 h. **(C)** Metabolic viability of planktonic and biofilm cells at different concentrations (25–400 μg mL^-1^). Error bars indicate standard deviations. Statistical significance was determined using one way ANOVA followed by Duncan *post hoc* test. Single asterisk represents statistical significance *p* < 0.05.

*S. cumini* methanolic exhibited concentration dependent antibiofilm activity against *C. albicans.* A maximum of 85% of biofilm inhibition was observed at 400 μg mL^-1^ concentration and at increasing concentrations, no further significant increase in biofilm inhibition was noticed. Thus, 400 μg mL^-1^ was considered as BIC of SCME against *C. albicans.* The cell density (absorbance at 600 nm) was similar at all the tested concentrations (Figure 1A). Further, non-fungicidal effect of SCME was confirmed by growth curve analysis. Thus, SCME at BIC does not affect the growth of *C. albicans* up to 24 h (Figure 1B). These results was further supported by XTT assay, as shown in Figure 1C wherein, SCME decreased the cell viability of *C. albicans* biofilm cells in dose dependent manner and whereas the viability of planktonic cells was similar to that of control.

### Purification and Characterization of Bioactive Principle From SCME

Antibiofilm activity of column fractions against *C. albicans* was assessed by crystal violet quantification method. Among the tested fractions, Ethylacetate80:Methanol20 (EA80:MET20) fraction showed maximum biofilm inhibition upto 75% without any fungicidal effect (Supplementary Figure S1). This fraction was further subjected to GC-MS analysis and the compounds present in active fraction are listed in Supplementary Table S2. Phenol, 4,4′-(1-methylethylidene)bis[2-methyl] (28.6%), (-)-quininic acid (14.81%), Isovaleric acid, nonyl ester (8.78%) and 4,5,7-trihydroxy-2-Octenoic acid (6.77%) were the major compounds identified from GC-MS analysis (Supplementary Figure S2).

### Effect of QA and UDA and Their Combination on Growth and Biofilm Formation

#### Effect of QA and UDA Combination on *Candida* spp. Growth

Quinic acid did not inhibit the growth of all the tested *Candida* strains at varying concentrations (12.5–800 μg mL^-1^). Conversely, MIC of UDA against wild type *C. albicans* (ATCC), *C. albicans* (MTCC), *C. glabrata* (MTCC), *C. tropicalis* (MTCC), *C. albicans* clinical isolate CA2, and *C. tropicalis* clinical isolates CT1, CT2, CT3 was found to be 160 μg mL^-1^. The MIC of UDA against *C. albicans* clinical isolates CA1, CA3, CA4 was found to be 80 μg mL^-1^ (Supplementary Figure S3).

#### Effect of QA and UDA Combination on *Candida* spp. Biofilm

**FIGURE 2:**
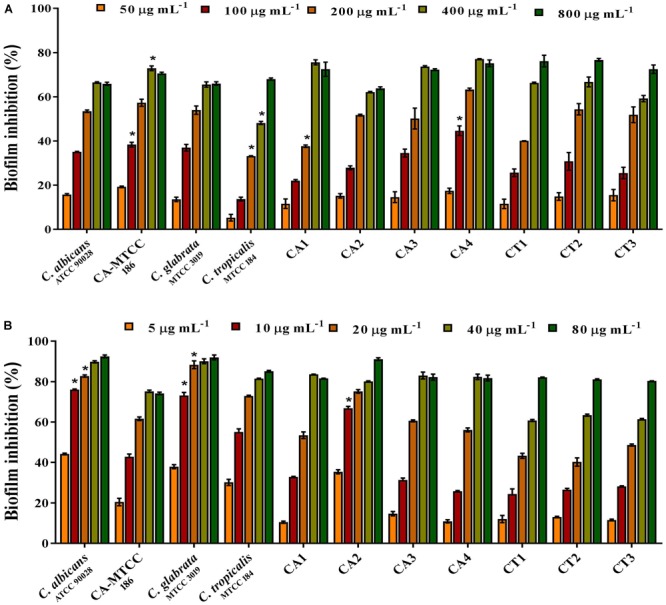
Effect of QA and UDA alone in *Candida* spp. biofilm. Antibiofilm potential of **(A)** QA at the concentrations from 50 to 800 μg mL^-1^. **(B)** UDA at the concentrations from 5 to 80 μg mL^-1^. Error bars indicate standard deviations. Single asterisk represents statistical significance *p* < 0.05.

The results showed that individual BIC of QA and UDA was varying between *Candida* species. BIC of QA against wild type *C. albicans* (ATCC), *C. albicans* (MTCC), *C. glabrata* (MTCC), and *C. albicans* clinical isolates CA1, CA2, CA3, and CA4 was found to be 400 μg mL^-1^ and the biofilm inhibition ranged between 65 and 77%. Further, QA inhibited the biofilms of *C. tropicalis* (MTCC) and the clinical isolates CT1, CT2, and CT3 in the range of 68–76% at 800 μg mL^-1^ (Figure 2A). Similarly, BIC of UDA against *C. albicans* (ATCC) and *C. albicans* (MTCC), *C. glabrata* (MTCC), *C. albicans* clinical isolates CA1, CA2, CA3, CA4 were identified as 20 and 40 μg mL^-1^, respectively. Biofilm was inhibited in the range of 75–91% upon treatment with UDA. Whereas in *C. tropicalis* (MTCC) and the clinical isolates CT1, CT2, and CT3, maximum biofilm reduction (>80%) was noticed at 80 μg mL^-1^ of UDA. Hence, 80 μg mL^-1^ concentration was considered as BIC (Figure 2B).

#### Combinatorial Effect of QA and UDA Against *Candida* spp. Biofilm

**FIGURE 3:**
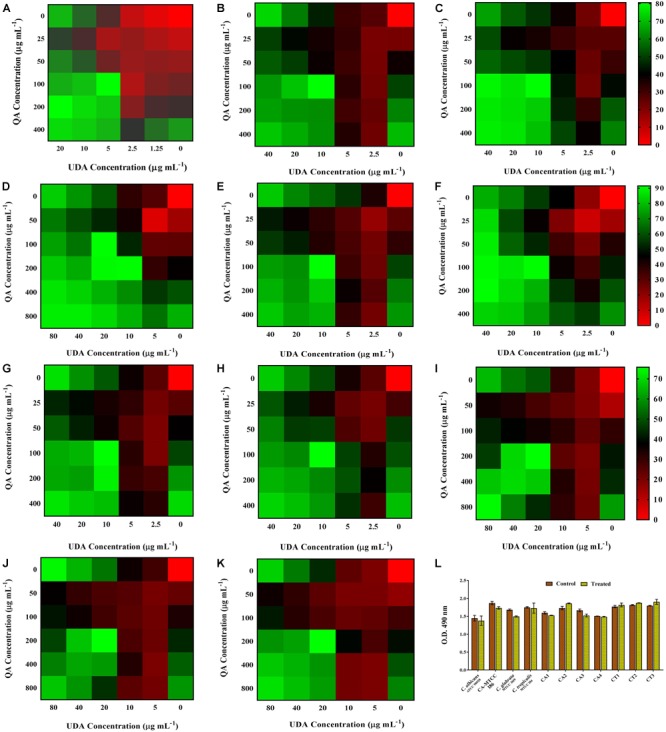
Effect of QA-UDA combinations (at BIC) against *Candida* spp. biofilm. **(A)** *C. albicans* (ATCC 90028), **(B)** *C. albicans* (MTCC 186), **(C)** *C. glabrata* (MTCC 3019), **(D)** *C. tropicalis* (MTCC 184), **(E)** *C. albicans* clinical isolates CA1, **(F)** CA2, **(G)** CA3, **(H)** CA4, **(I)** *C. tropicalis* clinical isolate CT1, **(J)** CT2, **(K)** CT3. In the graphs, red, blue, and green colors represent low (0–25%), moderate (25–50%), and high (50–80%) biofilm inhibition of *Candida* spp., respectively. **(L)** Effect of QA-UDA combination (at BIC) on the viability of *Candida* spp. No statistically significant change in the cell viability of *Candida* spp. was observed in the presence of QA-UDA combination when compared to untreated control. Error bars indicate standard deviations.

To evaluate the synergistic effect of QA-UDA combination against *Candida* spp., checkerboard assay was performed and the FICI values were calculated for each organism. BIC of QA and UDA in combination against *C. albicans* was found to be 100 and 5 μg mL^-1^, respectively. Whereas, BIC of QA and UDA against CA-MTCC, *C. glabrata* and *C. albicans* clinical isolates CA1, CA2, CA3, and CA4 were found to be 100 and 10 μg mL^-1^, respectively. On the other hand, *C. tropicalis* and the clinical isolates CT1, CT2, and CT3 showed maximum inhibition at the concentration of 200 and 20 μg mL^-1^ of QA and UDA, respectively. The level of biofilm inhibition was determined to be in the range of 72–91% (Figure 3). BIC of QA and UDA in combination was used for further experiments. FICI values of QA-UDA combination were found to be 0.375 and 0.5 which clearly revealed the synergistic antibiofilm activity against the tested *Candida* strains. FICI values are summarized in Table 2.

**Table 2:** Biofilm inhibitory concentrations of QA and UDA (individual or in combination) against *Candida* spp.

*Candida* strain	BIC of individual Compound (μg mL^-1^)		BIC of compounds in combination (μg mL^-1^)		FICI
	QA	UDA	QA	UDA	
*C. albicans* ATCC 90028	400	20	100	5	0.5–1.0
*C. albicans* MTCC 186	400	40	100	10	0.5–1.0
*C. glabrata* MTCC 3019	400	40	100	10	0.5–1.0
*C. tropicalis* MTCC 184	800	80	200	20	0.375–1.0
CA1	400	40	100	10	0.5–1.0
CA2	400	40	100	10	0.375–1.0
CA3	400	40	100	10	0.5–1.0
CA4	400	40	100	10	0.5–1.0
CT1	800	80	200	20	0.5–1.0
CT2	800	80	200	20	0.5–1.0
CT3	800	80	200	20	0.5–1.0

*BIC, biofilm inhibitory concentration; FICI, fractional inhibitory concentration index; QA, quinic acid; UDA, undecanoic acid. QA-UDA combination has a significant reduction in BIC than QA or UDA alone by Student’s t-test (p < 0.005).*

#### Effect of QA-UDA Combination on *Candida* spp. Cell Viability

XTT assay was performed to assess the non-fungicidal antibiofilm effect of QA-UDA combination against all the tested *Candida* strains. As shown in Figure 3L, the viability of treated cells was similar to that of control samples in all the tested strains. This result clearly confirmed that QA-UDA combination did not inhibit the metabolic viability of *Candida* spp.

#### Microscopic Examination of Mature Biofilm Architecture

##### Effect of QA-UDA combination on microcolony formation

**FIGURE 4:**
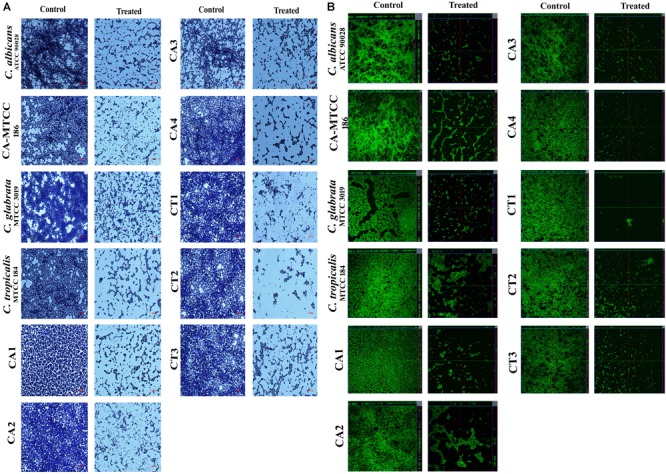
Microscopic images of *Candida* spp. Biofilm. **(A)** Light micrographs representing inhibitory effect of QA-UDA combination on microcolony formation of biofilm at the magnification of 400×. **(B)** CLSM images depicting the QA-UDA combinatorial effect of inhibition and biofilm architecture and hyphal elongation. Scale bar – 50 μm.

To confirm antibiofilm potential of QA-UDA combinations on glass surface, light microscopic analysis was performed. In the light micrographs, control samples were covered with thick layer of well-structured biofilm matrix with yeast and hyphae cells. In contrast, in treated samples, microcolony formation and hyphal elongation were completely inhibited by synergistic combinations of QA and UDA (Figure 4A).

##### Effect of QA-UDA on mature biofilm architecture

To further substantiate the antibiofilm potential of QA-UDA combination, CLSM analysis was performed. In all the tested strains, synergistic combination of QA-UDA significantly reduced the mature biofilm architecture (Figure 4B). In addition, COMSTAT analysis was carried out to measure the biomass, biofilm thickness and surface to volume ratio. Results clearly indicated the substantial reduction in all the tested parameters upon treatment with QA-UDA combination (Table 3).

**Table 3:** COMSTAT analysis of *Candida* spp. biofilm.

*Candid*a strain	Biomass (μm)		Maximum thickness (μm)		Surface - volume ratio (μm^2^/μm^3^)	
	Control	Treated	Control	Treated	Control	Treated
*C. albicans* ATCC 90028	43.05 ± 1.01	31.71 ± 1.04	41 ± 0.75	30.2 ± 1.09	0.02793 ± 0.001	0.0364 ± 0.000
*C. albicans* MTCC 186	26.67 ± 0.70	21.56 ± 1.24	25.4 ± 0.69	20.58 ± 0.81	0.04 ± 0.002	0.049 ± 0.003
*C. glabrata* MTCC 3019	38.98 ± 1.25	23.782 ± 1.14	36.69 ± 1.26	22.65 ± 0.66	0.03 ± 0.000	0.046 ± 0.001
*C. tropicalis* MTCC 184	33.22 ± 0.62	31.71 ± 0.75	31.71 ± 0.69	30.2 ± 1.15	0.03481 ± 0.000	0.0362 ± 0.000
CA1	28.98 ± 0.93	21.84 ± 0.49	27.6 ± 1.55	20.8 ± 0.40	0.0373 ± 0.000	0.04861 ± 0.000
CA2	31.7 ± 0.80	30.26 ± 0.86	30.2 ± 1.03	28.8 ± 0.86	0.036 ± 0.001	0.0377 ± 0.000
CA3	29.4 ± 1.09	21.84 ± 0.73	28 ± 0.63	20 ± 0.98	0.0368 ± 0.000	0.4481 ± 0.000
CA4	26.6 ± 1.50	20.58 ± 1.24	25.2 ± 0.69	19.6 ± 0.69	0.0404 ± 0.000	0.05141 ± 0.001
CT1	26.25 ± 0.37	25 ± 0.70	25 ± 0.28	23.75 ± 0.77	0.04139 ± 0.000	0.04282 ± 0.000
CT2	22.26 ± 0.10	18.9 ± 0.83	21.2 ± 0.63	18 ± 0.57	0.04775 ± 0.002	0.5573 ± 0.000
CT3	28.35 ± 0.71	24.15 ± 0.83	27 ± 0.23	23 ± 0.31	0.0381 ± 0.000	0.04423 ± 0.000

*Mean values of three independent experiments and Standard deviations are represented.*

### Effect of QA-UDA on EPS Production

#### FTIR Analysis

**FIGURE 5:**
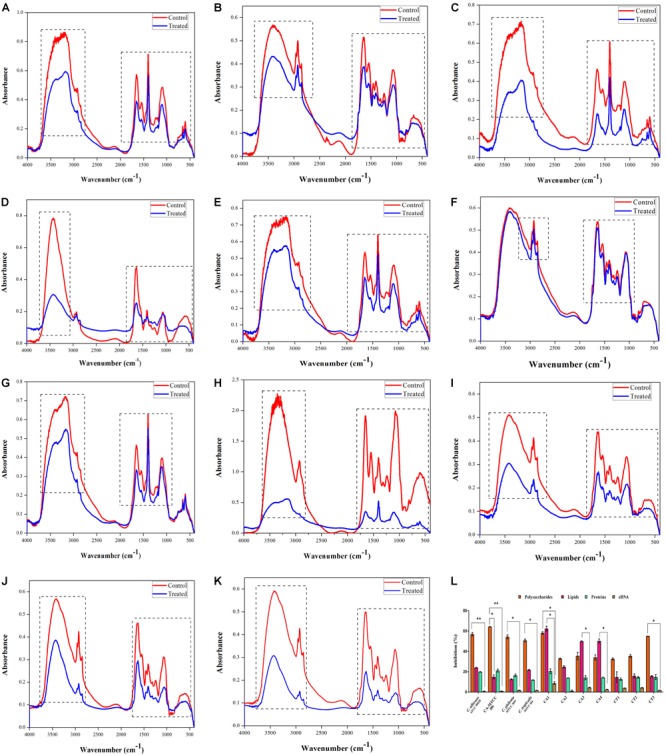
Inhibitory effect of QA-UDA combination on EPS of *Candida* spp. FTIR analysis of EPS extracted from *Candida* spp. in the absence and presence of QA-UDA combination. Dashed rectangles depict variation in the regions of 3600–3100 cm^-1^, 3000–2800 cm^-1^, 1700–1000 cm^-1^, and 700–500 cm^-1^ corresponding to the EPS components. **(A)** *C. albicans* (ATCC 90028), **(B)** *C. albicans* (MTCC 186), **(C)** *C. glabrata* (MTCC 3019), **(D)** *C. tropicalis* (MTCC 184), **(E)** *C. albicans* clinical isolates CA1, **(F)** CA2, **(G)** CA3, **(H)** CA4, **(I)** *C. tropicalis* clinical isolate CT1, **(J)** CT2, **(K)** CT3. **(L)** Bar graph representing the inhibitory effect of QA-UDA combination on polysaccharides, lipids, proteins, and eDNA present in the EPS. Error bars indicate standard deviations. A single and double asterisk(s) indicate the statistical significance between EPS components and the *p*-values are 0.05 and 0.01, respectively.

To study the effect of QA-UDA combination on *Candida* spp., EPS was analyzed by FTIR. FTIR spectra of all the tested strains exhibited major peaks at the regions of 3600–3100 cm^-1^, 3000–2800 cm^-1^, 1700–1000 cm^-1^, and 700–500 cm^-1^ which correspond to hydroxyl group of polysaccharides, asymmetrical C-H stretching vibration of aliphatic CH_2_, amide II stretching vibration, stretching vibration of COO– functional groups of amino acid side chains of free fatty acids, *O*-acetyl ester linkage bonds of uronic acid and C-X stretch of alkyl halides (Figure 5). In comparison with control spectra, QA-UDA treated spectra showed significant changes in the peak height and intensity which are directly correlated to considerable changes in polysaccharides, fatty acids and proteins present in the EPS of *Candida* spp.

#### Quantification of EPS Components

The EPS components such as polysaccharides, lipids, protein and eDNA play a vital role in maintaining the integrity of biofilms. The phenol sulfuric acid quantification of total carbohydrates clearly showed that QA-UDA combination significantly reduced the polysaccharides level in the range of 32–64%. Lipid content was also decreased up to 62, 50, and 49% in CA1, CA4, and CA3, respectively; whereas in all other *Candida* strains the inhibition level ranged between 12 and 23%. On the other hand, no considerable change was observed in protein and eDNA content of EPS upon treatment with QA-UDA combination (Figure 5).

### Effect of QA-UDA on Filamentous Growth

**FIGURE 6:**
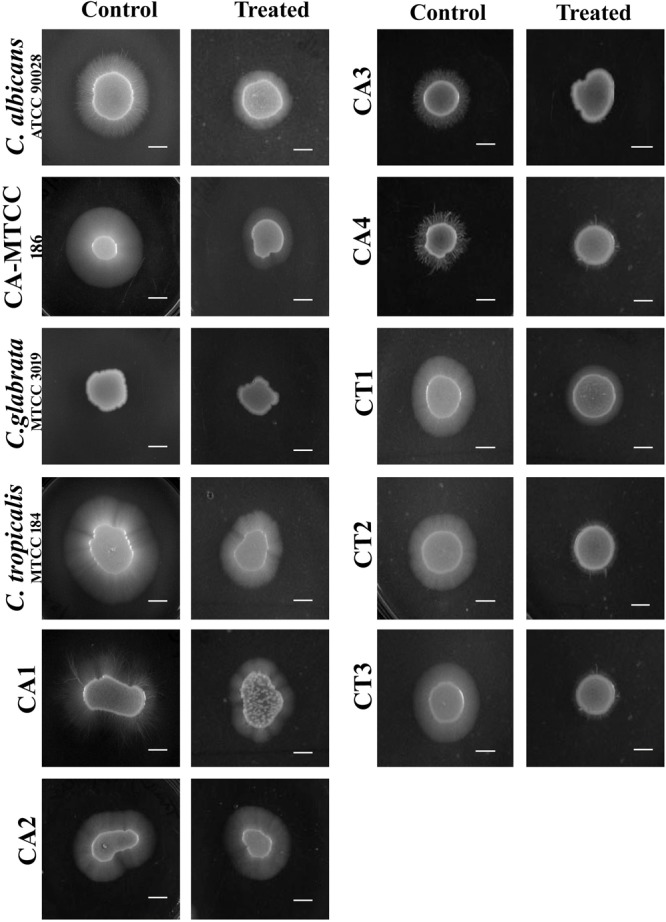
Inhibition of *Candida* spp. filamentous growth in the absence and presence of QA-UDA combination in spider agar supplemented with 10% FBS. Reduced filamentous growth was observed in QA-UDA combinations treated *Candida* spp. Scale bar – 0.5 cm.

The inhibitory effect of QA-UDA combinations on *Candida* spp. filamentation was assessed using spider agar supplemented with FBS. In the reference strains of *C. albicans* (ATCC and MTCC) and the clinical isolates (CA1, CA2, CA3, and CA4), filamentous growth was significantly inhibited by QA-UDA combination. However, moderate inhibition was observed in *C. tropicalis* (MTCC) and its isolates (CT1, CT2, and CT3). No filamentous growth was observed in *C. glabrata* even after 72 h of incubation (Figure 6).

### Effect of QA-UDA on Hydrolases and Ergosterol Production

#### Secreted Aspartyl Proteinases (SAPs)

**FIGURE 7:**
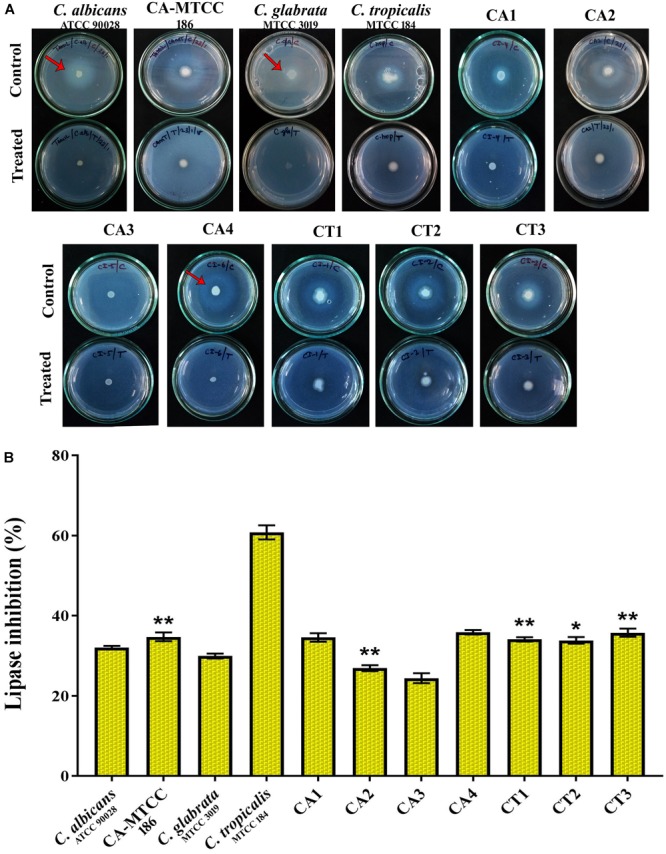
**(A)** Effect of QA-UDA combinations on SAPs production by *Candida* spp. Compared to control plates, SAPs production was drastically reduced upon treatment with QA-UDA combination by means of vanished white opaque zone around the colony. White precipitation zone around the colonies are specified by red arrow. **(B)** Effect of QA-UDA combinations on lipases production by *Candida* spp. Bar graph represents percentage inhibition of lipase production in the tested *Candida* strains. Error bars indicate standard deviations. A single and double asterisk(s) indicate the statistical significance and the *p*-values are 0.05 and 0.01, respectively.

Secreted aspartyl proteinases production was qualitatively assessed by the white opaque zone around the colonies. In all the 11 *Candida* strains, SAPs production was significantly inhibited by QA-UDA combinations compared to respective controls (Figure 7A).

#### Phospholipase and Lipases

*Candida albicans* (ATCC) and the clinical isolates CA1, CA3, and CA4 only showed precipitation zone around colonies known as phospholipases production which was significantly inhibited by QA-UDA combinations (Table 4). No phospholipase production was noticed in *C. albicans* (MTCC), and the Clinical isolates CA2, CT1, CT2, and CT3. Besides, lipase production was evaluated by measuring the zone of clearance around the colonies and the zone diameter was calculated for both control and treated plates (Table 4). The qualitative measurement of lipase production in *C. albicans* (ATCC), *C. albicans* (MTCC), *C. glabrata* (MTCC), CA1, CT2, and CT3 showed slight inhibition by QA-UDA combination compared to untreated control. In other *Candida* strains, zone of inhibition was found to be equal in both control and treated plates. On the other hand, quantitative measurement of lipase production clearly revealed significant inhibition (in the range of 26–60%) upon treatment with QA-UDA combination (Figure 7B).

**Table 4:** Effect of combination of QA and UDA on *Candida* spp. phospholipase and lipase production.

*Candida* strain	Phospholipase production		Lipase production Zone of inhibition (mm)	
	Control	Treated	Control	Treated
*C. albicans* ATCC 90028	+++	+	28.25 ± 2.95	26.50 ± 3.34
*C. albicans* MTCC 186	**–**	**–**	35.50 ± 1.54	33.25 ± 3.26
*C. glabrata* MTCC 3019	**–**	**–**	29.00 ± 1.63	27.00 ± 2.30
*C. tropicalis* MTCC 184	**–**	**–**	30.25 ± 1.52	29.75 ± 2.01
CA1	++	+	27.50 ± 1.78	25.75 ± 3.01
CA2	**–**	**–**	32.00 ± 3.18	31.25 ± 3.48
CA3	++	+	31.00 ± 1.26	29.25 ± 2.41
CA4	++	+	27.75 ± 1.34	27.75 ± 2.18
CT1	**–**	**–**	29.25 ± 3.75	29.25 ± 2.98
CT2	**–**	**–**	30.25 ± 3.13	28.25 ± 3.18
CT3	**–**	**–**	30.50 ± 3.01	28.75 ± 3.75

*‘+++’, strong activity; ‘++’, moderate activity; ‘+’, low activity; ‘-’, no activity. Mean values of three independent experiments and Standard deviations are represented*.

#### Ergosterol Production

**FIGURE 8:**
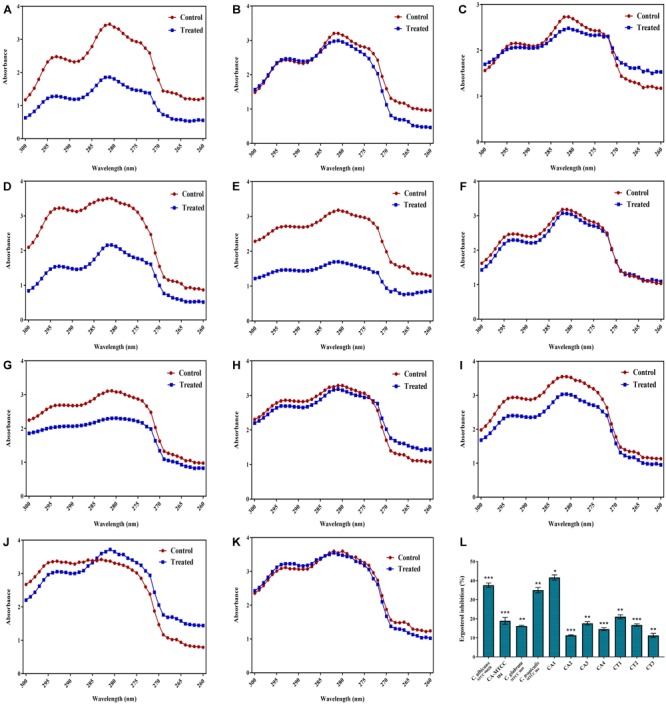
Efficacy of QA-UDA combination on Ergosterol production. Ergosterol profile of *Candida* spp. in the presence and absence of QA-UDA combination scanned between 260 and 300 nm. Reduction in the peak height represents changes in ergosterol content. **(A)** *C. albicans* (ATCC 90028), **(B)** *C. albicans* (MTCC 186), **(C)** *C. glabrata* (MTCC 3019), **(D)** *C. tropicalis* (MTCC 184), **(E)** *C. albicans* clinical isolates CA1, **(F)** CA2, **(G)** CA3, **(H)** CA4, **(I)** *C. tropicalis* clinical isolate CT1, **(J)** CT2, **(K)** CT3. **(L)** Percentage inhibition of total sterol content of *Candida* spp. Error bars indicate standard deviations. A single, double, and triple asterisk(s) indicate the statistical significance and the *p*-values are 0.05, 0.01 and 0.001, respectively.

As change in sterol content can alter the membrane functions of *Candida* spp., effect of QA-UDA combination on ergosterol composition was evaluated using UV Spectrophotometer. In the UV-spectra, presence of peaks observed between 260 and 300 nm represented the ergosterol and sterol intermediates. In QA-UDA treated samples of *C. albicans, C. tropicalis*, CA1, CA3, CT1, and CT2, characteristic peaks representing ergosterol were significantly reduced by combination of QA-UDA. However, slight changes were observed in the sterol content of *C. glabrata*, CA2, CA4, and CT3 upon treatment with QA-UDA combination (Figure 8). In addition, ergosterols were quantified by spectrometric method and the percentage of inhibition was calculated. A maximum of 41, 37, and 35% ergosterol inhibition was observed in CA1, *C. albicans* and *C. tropicalis*, respectively. In other strains, the inhibition level was in the range of 11–21% (Figure 8).

### Impact of QA-UDA Combination on *C. albicans* Gene Expression

**FIGURE 9:**
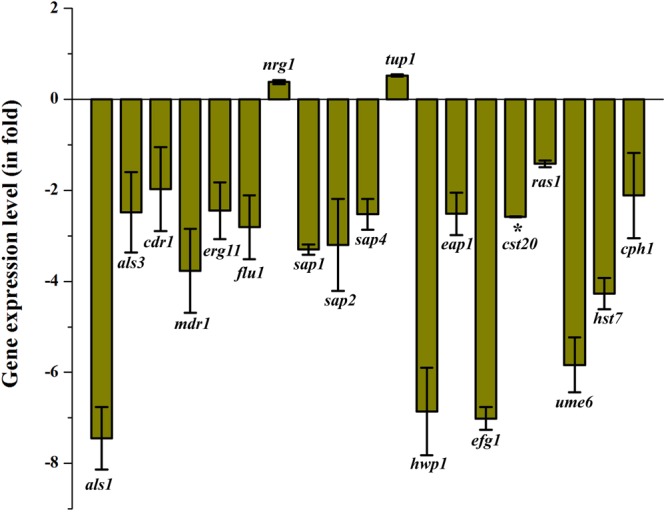
Gene expression analyses of *C. albicans* candidate virulence genes in the presence of QA-UDA combination. Relative gene expression level was determined using the ΔΔ*C*T method. Error bars indicate standard deviations from the mean (*n* = 2). Single asterisk represent statistical significance (*p* < 0.05).

The impact of QA-UDA combination on the *C. albicans* genes which are the key regulators of biofilm and virulence mechanism was evaluated using quantitative PCR analysis. Among the tested genes, *als1, hwp1, efg1*, and *ume6* were drastically down regulated to -7.4, -6.8, -7.0, and -5.8-fold, respectively. Moreover, *als3* (-2.4-fold), *cdr1* (-1.9), *mdr1* (-3.7-fold), *erg11* (-2.4-fold), *flu1* (-2.8-fold), *sap1* (-3.3-fold), *sap2* (-3.2), *sap4* (-2.5) fold, *eap1* (-2.5-fold), *cst20* (-2.5-fold), *ras1* (-1.4-fold), *hst7* (-4.2-fold) and *cph1* (-2.1-fold) genes were moderately down regulated upon treatment with QA-UDA combination. On the other hand, slight up regulation was observed in *nrg1* and *tup1* up to 0.3- and 0.5-fold, respectively (Figure 9).

### Effect of QA-UDA Combination on *in vivo* Biofilm and Virulence

**FIGURE 10:**
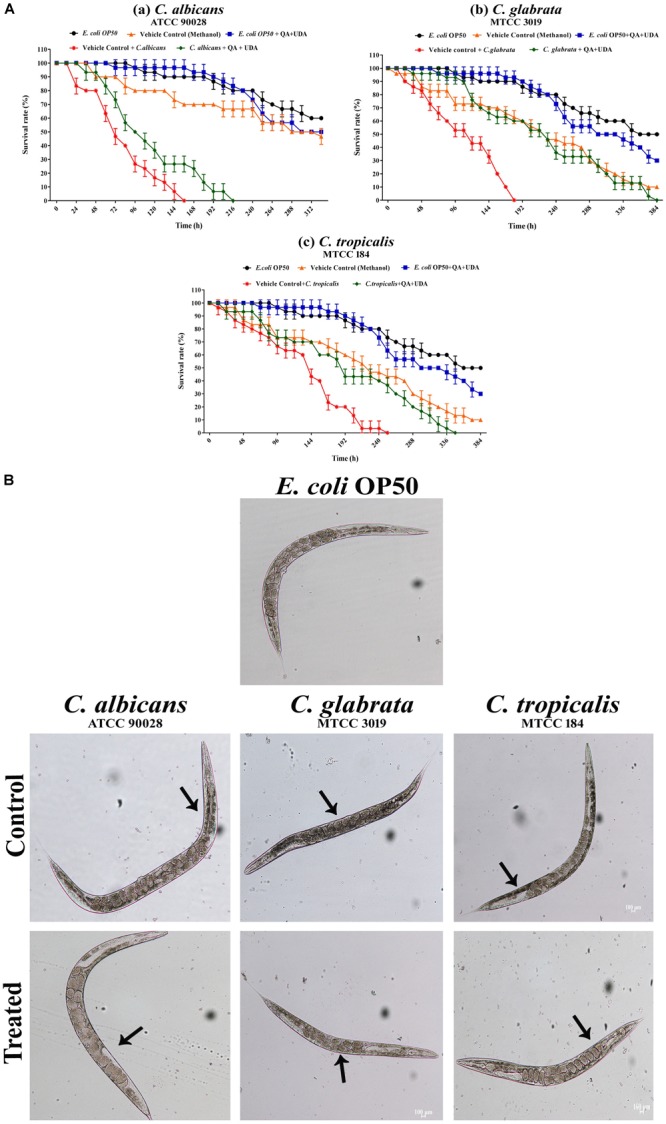
Effect of QA-UDA combination on *Candida* spp. *in vivo* biofilm formation. **(A)** Survival assay of *C. albicans* **(a)**, *C. glabrata* **(b),** and *C. tropicalis* **(c)** demonstrating insignificant difference observed between QA-UDA treated groups and *E. coli* OP50 (food source) groups proving the non-toxic nature of QA-UDA. **(B)** Light microscopic images illustrate the reduced internal colonization of *Candida* spp. in the presence of QA-UDA than their respective controls. Magnification 400×, scale bar – 100 μm.

To assess the cytotoxic nature of QA-UDA combination, *C. elegans* survival assay was performed. In all the tested strains, no significant change was observed in two groups viz. *E. coli* OP50 and *E. coli* OP50+QA-UDA (Figure 10A). This result clearly proved the non-toxic nature of QA-UDA combination. Similarly, vehicle control methanol was also not lethal to worms. In addition, impact of QA-UDA combination on *in vivo* biofilm and virulence of *Candida* spp. were also evaluated. The worms exposed to *C. albicans, C. glabrata*, and *C. tropicalis* exhibited survival rate of 156, 180, and 252 h, respectively. At the same time, the worms exposed to *C. albicans, C. glabrata*, and *C. tropicalis* along with QA-UDA combination showed increased survival rate of 216, 384, and 348 h, respectively. In the light micrograph, visible colonization was observed in control worms, whereas in QA-UDA combination treated worms displayed considerably reduced internal colonization (Figure 10B). This result confirmed that the QA-UDA combination reduced the *in vivo* biofilm and virulence of *Candida* spp.

## Discussion

*Candida* spp. is proficient in forming biofilm on both medical implants and body surfaces (Mayer et al., 2013). For successful treatment and control the root of the infection, suitable antifungal therapy is needed. Antifungal resistance is the decisive problem remain to be solved and to circumvent these issues, comprehensive research about alternative therapeutic agents and techniques are required. In this perspective, the present study demonstrated the synergistic antibiofilm activity of *S. cumini* derived quinic acid and undecanoic acid against *Candida* spp.

Initially, the antibiofilm and antifungal effect of SCME was evaluated using crystal violet quantification assay and growth curve analysis, respectively. The results clearly suggested that SCME showed non-fungicidal antibiofilm activity against *C. albicans.* The *S. cumini* plant already has been reported for its antioxidant, antimicrobial, and antibiofilm properties against various bacterial pathogens (Ruan et al., 2008; Mohamed et al., 2013; Gopu et al., 2015). GC-MS analysis of active leads disclosed the presence of quinic acid as one of its major compounds. Whereas, amides of QA have been reported for their antimicrobial activity against both Gram-positive and Gram-negative bacterial pathogens (Rezende et al., 2015). Additionally, QA with other phenolic acids have shown significant antiviral, antibacterial, antifungal activity and the non-toxic nature of QA was confirmed by Madin-Darby bovine kidney and Vero cell lines (Özçelik et al., 2011). QA derivative (5-*O*-caffeoyl quinic acid) has already been shown to possess antifungal activity against *Aspergillus* spp. (Suárez-Quiroz et al., 2013). For centuries, fatty acids are known for their antifungal properties. UDA is a cost-effective antifungal compound, used as an active ingredient of topical antifungal formulations and the mechanism responsible for its antifungal effect is inhibition of yeast-to-hyphal transition and fatty acid biosynthesis (Prince, 1959; Anon, 2002). Besides, Li et al. (2008), demonstrated that the acetylenic acid including UDA exhibited significant antifungal potencies against various fungal pathogens such as *Candida* spp., *Aspergillus* spp., *Trichophyton* spp., and *Cryptococcus neoformans* and also confirmed the *in vitro* and *in vivo* non-toxic effect UDA. Similarly, in, Shi et al. (2016) divulged the possible mechanism of action that UDA targets *Candida* spp., virulence traits such as hyphal formation, adhesion, mitochondrial activity, cell proliferation, transcriptional regulation of the cell membrane formation and biofilm formation. Moreover, recent study of UDA loaded hexosomes exhibited remarkable inhibition of *C. albicans* growth and filamentation with no adverse effect in human cells (Mionić Ebersold et al., 2018). However, inhibitory effect of QA and UDA against *Candida* spp. biofilm and virulence remains unexplored. Thus, the current study is intended to assess the combinatorial effect of quinic acid and undecanoic acid against wild type and clinical isolates of *Candida* spp. Initially, individual effect of QA and UDA against *Candida* spp. growth and biofilm was assessed. QA and UDA exhibited non-fungicidal antibiofilm activity at tested concentration (BIC). Through the checkerboard experiment, FICI value of QA-UDA combination was determined as ≤0.5, evidencing that QA and UDA synergistically inhibited biofilm formation in *Candida* spp. Likewise, Sharma et al. (2014) have reported the combinatorial antimicrobial activity of curcumin with selected phytochemicals against *Staphylococcus aureus*. Similarly, phytocompounds and antifungal agents combinations such as thymol with nystatin (de Castro et al., 2015), cinnamaldehyde, citral, eugenol, and geraniol with fluconazole (Khan and Ahmad, 2012), Thionin-like peptide from *Capsicum annuum* fruits with fluconazole (Taveira et al., 2016) exhibiting antifungal and antibiofilm efficiency against *Candida* spp. have been reported. In this background, to the best of investigators’ knowledge, the present study is the first attempt to explore the synergistic antibiofilm effect of the phytochemicals QA and UDA against *C. albicans, C. tropicalis*, and *C. glabrata.* According to literature, drugs or antibiotics which affect normal growth and viability of the pathogens, put an organism under selective pressure to develop resistance (Rasmussen and Givskov, 2006; Sethupathy et al., 2016). In the present study, XTT assay results revealed the QA-UDA synergistic combination displaying non-fungicidal antibiofilm potential against the tested *Candida* strains. Thus, the chance of getting drug resistance is very meager. Further, Light and CLSM micrographs visibly evidenced that hyphal elongation, microcolony formation, biofilm thickness were considerably decreased by QA-UDA combination. In the same way, usnic acid and 3,5-Di-tert-butylphenol diminished the hyphal growth and biofilm thickness of *C. albicans* and *C. tropicalis* (Nithyanand et al., 2015; Rathna et al., 2016).

Although the pathogenicity and virulence of each *Candida* species vary between species, the major virulence factors of *Candida* spp. include adhesion and invasion on host tissue, biofilm formation, phenotypic switching, filamentous growth, production of extracellular enzymes and EPS (Inci et al., 2012; Muthamil and Pandian, 2016). Cells present in the biofilm are surrounded by the hydrated matrix known as EPS which provides complex, three dimensional structures to the biofilm and thwarts the penetration of antifungal agents (Sauer et al., 2007; Shafreen et al., 2014). FTIR analysis of the current study confirmed that the QA-UDA combination noticeably altered the polysaccharides, amino acids, fatty acids contents of EPS. Besides, spectrophotometric quantification of EPS components also substantiated the effect of QA-UDA combination on EPS inhibition, albeit insignificant changes observed in eDNA and protein contents of EPS upon treatment with QA-UDA combination. In comparison, essential oils from *Pogostemon heyneanus, Cinnamomum tamala*, and *Cinnamomum camphora* considerably disrupted the extracellular matrix of *C. albicans, C. tropicalis*, and *C. glabrata* (Banu et al., 2018).

Most of the fungal pathogens undergo morphological changes at some point of host invasion. For example, yeast cells of *Candida* species form filamentous hyphae or pseudohyphae (Gow et al., 2002). This phenotypic switching in *Candida* species is controlled by many environmental cues such as increased extracellular pH, starvation of glucose, deficiency of O_2_, elevated CO_2_, *N*-acetylglucosamine, amino acids and nitrogen starvation (Csank and Haynes, 2000; Morales et al., 2013; Muthamil and Pandian, 2016). From the results of filamentation assay, it is clear that QA-UDA combination profoundly inhibited the filamentous growth of *C. albicans* on solid agar medium, while slightly inhibited filamentous colony was observed in both wild type and clinical isolates of *C. tropicalis.* This is in line with the results wherein, small molecules such as diazaspiro-decane structural analogs and filastatin showed strong inhibition against filamentous growth of *C. albicans* (Pierce et al., 2015; Vila et al., 2017).

In *C. albicans*, SAPs known as sap protein family (Sap 1–10) are involved in the maintenance of cell wall integrity and regulation of attachment of *C. albicans* with human epithelial cells and neutrophils (Buu and Chen, 2013). Other than *C. albicans*, some NAC species including *C. dubliniensis, C. tropicalis, C. parapsilosis*, and *C. glabrata* are also capable of producing SAPs (Naglik et al., 2003; Sikora et al., 2011). In this study, qualitative measurement of SAPs production in all the tested strains revealed the intense reduction upon treatment with QA-UDA combinations. Other than SAPs, lipolytic enzymes such as phospholipases and lipases contribute to virulence of pathogenic *Candida* spp. including morphological changes, diffusion to the host, colonization and cytotoxicity (Park et al., 2013). *C. albicans* contains approximately 10 lipase coding genes and 5 phospholipases classes. However, lipases and phospholipases are considered as one among the virulence factors of NAC species (Kantarcioǧlu and Yücel, 2002; Zuza-Alves et al., 2017). In this study, phospholipases production was noticed in *C. albicans* (ATCC) and in some of the clinical isolates (CA1, CA3, and CA4) but not in *C. glabrata, C. tropicalis*. At the same time, lipase production was observed in all the 11 tested strains. QA-UDA combination slightly affected the lipase production in the tested *Candida* strains. In a similar way, AgNPs from *Dodonaea viscosa* and *Hyptis suaveolens* plants and 2,4-di-tert-butyl phenol moderately inhibited the SAPs production in *Candida* spp. (Padmavathi et al., 2015b; Muthamil et al., 2018). Similarly, 5-hydroxymethyl-2-furaldehyde from marine bacterium considerably reduced the SAPs and phospholipases of *C. albicans* (Subramenium et al., 2018). Ergosterol, a major component present in fungal cell membrane coordinates membrane heterogeneity, controls water penetration, maintain the integrity, fluidity of plasma membrane and regulates the enzymes involved in protein transport and chitin synthesis (Abe et al., 2009; Lv et al., 2016; Zuza-Alves et al., 2017). Of note, most of the antifungal agents used for the treatment of candidiasis, targets the enzymes involved in the ergosterol biosynthetic pathway (Onyewu et al., 2003; Shafreen et al., 2014). In the present study, reduced ergosterol content was observed in the presence of QA-UDA combination. This result in agreement with the observation made by Ahmad et al. (2010) wherein ergosterol synthesis was inhibited in *C. albicans* by eugenol and methyl eugenol in a dose dependent manner. Conversely, Xu et al. (2017) have reported that the berberine treatment in fluconazole-resistant *C. albicans* increased the ergosterol content and up regulated the genes involved in ergosterol biosynthesis.

To explicate the probable molecular mechanism exhibited by synergistic QA-UDA combination on *C. albicans*, gene expression profile of some candidate genes which are involved in biofilm and virulence were studied. Among the candidate genes analyzed, *als1* and *als3* were found to be evidently downregulated to -7.4- and -2.4-fold, respectively. This Als gene family codes for cell surface glucoproteins which are implicated in adhesion of *C. albicans* to various host surfaces (Hoyer, 2001; Green et al., 2006). Further, transcriptional analysis of both wild-type and hyphal defective mutants revealed that invasin-like protein Als3, is essential for ferritin binding in *C. albicans* (Almeida et al., 2008). In *C. albicans*, fluconazole resistance emerges through four possible mechanisms, first one is alteration in drug target enzyme such as point mutation in lanosterol 14-α demethylase gene (*erg11*), second mechanism is increased expression of genes encoding membrane transporters such as ATP-binding cassette (ABC) family (Cdr1, Cdr2) and major facilitator super family (MFS) (Mdr1, Flu1), third and fourth explanation for azole resistance in *C. albicans* might be considered as biofilm formation and vesicular vacuoles development, respectively (Chen et al., 2010; Rad et al., 2016). Thus, treatment of QA-UDA combination down regulating the expression of membrane transporter genes (*cdr1, mdr1*, and *flu1*) and lanosterol 14-α demethylase gene (*erg11*) in ergosterol pathway suggested that the probability of getting drug resistance can be eliminated. The down regulation of *erg11* genes reflected in spectrophotometric analysis of ergosterol further confirms the effect of QA-UDA combination ergosterol biosynthesis. This result is also in accordance with an earlier report in which levofloxacin derivatives and 5-hydroxymethyl-2-furaldehyde downregulated the *erg11* gene (Shafreen et al., 2014; Subramenium et al., 2018). SAPs have been recognized as one of the virulence factors of *Candida* spp. particularly, in *C. albicans*, ten aspartyl proteinases (Sap1–Sap10) facilitate the active penetration into the host cells (Smolenski et al., 1997; Sikora et al., 2011). Among the 10 Saps, Sap1–Sap8 secretes and diffuses into surrounding medium, while Sap9 and Sap10 attaches to the cell surface (Mayer et al., 2013). Accordingly, down regulation of *sap1, sap2*, and *sap4* genes resulted in remarkable inhibition of extracellular SAPs production in QA-UDA treated *C. albicans* in BSA agar medium. It is noteworthy to mention here that engineered antifungal peptide histatin-5 altered the proteolysis activity of Sap2 and Sap9 and enhanced the antifungal activity (Ikonomova et al., 2018). Similarly, dermaseptin-S1, another antimicrobial peptide, has been reported for its inhibitory effect on Saps (*sap1, sap2, sap3, sap9*, and *sap10*) along with growth, biofilm formation, morphological transition in *C. albicans* (Belmadani et al., 2018). Further, morphological transition from yeast to hyphae is one of the important virulence factors of *C. albicans.* Hwp1 is a major hyphal regulon that encodes Hypha-specific cell wall proteins entailed in host tissue penetration (Kumamoto and Vinces, 2005). In immunocompromised mice model, *hwp1* mutant strain failed to adhere on epithelial cells and defective in causing oral infection (Sundstrom et al., 2002). Moreover, hyphal specific gene expression was positively regulated by transcription factors such as Efg1, Cph1, Cph2, Tec1, Flo8, Czf1, Rim101, and Ndt 80 (Lane et al., 2001). Among these genes, Efg1 is based on cyclic AMP, considered to be major regulator of hyphal initiation which promotes hyphae in the presence of CO_2_, neutral pH, serum and *N*-acetylglucosamine in solid and liquid media. In contrast, Cph1 depends on mitogen-activated protein kinase (MAPK) signaling pathway triggered for hyphal formation only on solid medium (Spider agar) and not in liquid medium (Sudbery, 2011). Furthermore, either *efg1* or *cph1* deletion mutant showed reduced hyphal growth, substantially *efg1* and *cph1* double mutant of *C. albicans* failed to produce filamentation (Kumamoto and Vinces, 2005). (Carlisle and Kadosh, 2013), studied the upstream regulation of Ume6, an important filament specific transcriptional regulator, during sequential transition from yeast to pseudohyphae and/or hyphae. In *C. albicans*, Eap1 has been reported for its role in adhesion to the host as well as sensing of other organism on its surface (Fox et al., 2013). Ras proteins known as GTP-activating proteins (GTPases), conserved family of small eukaryotes, has been associated with controlling of cell shape, nutrient transport, stress response and mating (Inglis and Sherlock, 2013). Furthermore, Hst7 and Cst20 are homologs of Ste7 and Ste20 in *Saccharomyces cerevisiae*, based on MAPK cascade and known for their role in filamentation (Köhler and Fink, 1996). In this study, *hwp1, efg1, cph1, ume6, eap1, ras1, hst7*, and *cst20* genes that are directly or indirectly involved in hyphal growth or filamentation were found to be appreciably down regulated in the presence QA-UDA combination. Conversely, up regulation was noticed in *nrg1* and *tup1* genes and this results goes in parallel with the published literature wherein both Nrg1 and Tup1 are negative regulators of hyphal gene expression and filamentous growth (Kumamoto and Vinces, 2005; Sudbery, 2011; Goffena et al., 2018). Primitively, the toxic effect of QA-UDA combinations was analyzed using simple eukaryotic model organism, *C. elegans.* Accordingly, *C. elegans – Candida* infection most amenable model has been used for the high throughput screening of antifungal compounds against *Candida* spp. (Breger et al., 2007). Moreover, the nematode *C. elegans* conserves various biological functions of humans and also possesses orthologs of genes related to diseases. In addition, the nematode was used to study the microbial virulence factors including quorum sensing. *C. elegans* mutants can also be used for the better understanding of pathogenicity (Kaletta and Hengartner, 2006; Edwards and Kjellerup, 2012). From the results obtained from survival assay, it is apparent that QA-UDA combination does not exhibit toxicity to the worms. On the other hand, light micrographs suggested that *in vivo* colonization and virulence of *Candida* spp. was discernibly decreased by the treatment of QA-UDA combination.

## Conclusion

Overall, to the best of the investigators’ knowledge, the present study, for the first time, demonstrates the anti-virulent potential of *S. cumini* derived quinic acid in combination with the well-known antifungal agent undecanoic acid. The synergistic combination of quinic acid and undecanoic acid targets the genes involved in adhesion, morphological transition, ergosterol biosynthesis, secreted aspartyl proteinase and filamentation. These results reflected in the *in vitro* virulence assays as well. The inhibitory effect on *in vivo* biofilm formation and virulence was further confirmed using *C. elegans.* Though, further investigations in higher eukaryotic systems are needed for the suitable use of these phytochemicals, antivirulence potential from synergistic combination of phytochemicals might be a cost effective method than conventional antifungal therapy. Thus, the present study emphasizes synergism between quinic acid and undecanoic acid which in turn enhance their antipathogenic potential against biofilm related *Candida* infection.

## Author Contributions

SKP, SM, KB, and BB designed the experiments. SM and BB performed the experiments. SM analyzed the data. SM and SKP wrote the main manuscript. All authors approved the final manuscript.

## Conflict of Interest Statement

The authors declare that the research was conducted in the absence of any commercial or financial relationships that could be construed as a potential conflict of interest.
