# Supplementary material for: Synergistic Effect of Quinic Acid Derived From Syzygium cumini and Undecanoic Acid Against Candida spp. Biofilm and Virulence
Source: Front Microbiol. 2018 Nov 26;9:2835. doi: 10.3389/fmicb.2018.02835 (PMC6275436; doi:10.3389/fmicb.2018.02835)
Supplement: Supplementary file 1 [file Table_1.DOCX]

| **Gene** | **Function** | **Primer sequence (5’-3’)** | | **Reference** |
| --- | --- | --- | --- | --- |
|  |  | **Forward** | **Reverse** |  |
| *als1* | Adhesion, Agglutinin like protein | GCACCAATGTTGATGAGGTG | GGCAACAATTCCGAGCTTAC | Green et al. 2006 |
| *als3* | Adhesion, Agglutinin like protein | CAACTTGGGTTATTGAAACAAAAACA | AGAAACAGAAACCCAAGAACAACC | Green et al. 2006 |
| *cdr1* | ATP-binding cassette (ABC) superfamily, Multidrug transporter | ATGGCTATTGTTGAAACTGTCATTG | CCCTTTACCGAAAACTGGAGTAG | Liang et al. 2010 |
| *mdr1* | Major facilitators superfamily | TGTTGCCTTTGATGGTCCCG | ACCGATAAGGCAGCAAGACC | Karababa et al. 2004 |
| *erg11* | Ergosterol biosynthesis | GAAAGAGAACCATTACCAGG | AGGAATCGACGGATCAC | Zhou et al. 2018 |
| *flu1* | Fluconazole resistant gene | CACGTCTTTGTCGCAACAGC | ATGTTGTGACTTGCAGTAGC | Chen et al. 2010 |
| *nrg1* | Negative regulator of transcription | CCAAGTACCTCCACCAGCAT | GGGAGTTGGCCAGTAAATCA | Murad et al 2001  Kumamoto and Vinces 2005 |
| *sap1* | Secreted aspartyl proteinase | CAAGGTATCATGGGGATTGG | CAAAACAACATCAGCGTTGG | Liang et al. 2010 |
| *sap2* | Secreted aspartyl proteinase | GTTGGACTCTGGGACCACAT | CAAAGCACAGAAACCAGCAA | Liang et al. 2010 |
| *sap4* | Secreted aspartyl proteinase | AATTTACCGCCAACAAATCG | CCAATGGGTATTGGATCGAC | Liang et al. 2010 |
| *tup1* | Negative regulator of transcription | CTTGGAGTTGGCCCATAGAA | TGGTGCCACAATCTGTTGTT | Murad et al 2001 |
| *hwp1* | Hyphal wall protein  Adhesin | GCTCCTGCTCCTGAAATGAC | CTGGAGCAATTGGTGAGGTT | Carlisle and Kadosh, 2012; Kumamoto and Vinces 2005 |
| *eap1* | Adhesion | TGTGATGGCGGTTCTTGTTC | GGTAGTGACGGTGATGATAGTGACA | Fox et al. 2013 |
| *efg1* | Transcription factor | GCCTCGAGCACTTCCACTGT | TTTTTTCATCTTCCCACATGGTAGT | Kumamoto and Vinces 2005 |
| *cst20* | Filamentation | ATGTCTCATAATAATGGC | GGTTAATTAGTTTCTTC | Kohler and Fink 1996 |
| *ras1* | Cell adhesion, filamentous growth | CCCAACTATTGAGGATTCTTATCGTAAA | TCTCATGGCCAGATATTCTTCTTG | Inglis and asherlock 2013 |
| *ume6* | Transcriptional regulator of filamentous growth | ACCACCACTACCACCACCAC | TATCCCCATTTCCAAGTCCA | Carlisle and Kadosh, 2012 |
| *hst7* | Filamentation | TCATCAGCTTCTTCTATAC | TATTGAGGAAATGACAGTT | Kohler and Fink 1996 |
| *cph1* | Transcription factor | TATGACGCTTCTGGGTTTCC | ATCCCATGGCAATTTGTTGT | Kumamoto and Vinces 2005 |

**Supplementary Table 1.** List of genes, their role and primer sequences used in the study.
