# Supplementary material for: Synergistic Effect of Quinic Acid Derived From Syzygium cumini and Undecanoic Acid Against Candida spp. Biofilm and Virulence
Source: Front Microbiol. 2018 Nov 26;9:2835. doi: 10.3389/fmicb.2018.02835 (PMC6275436; doi:10.3389/fmicb.2018.02835)
Supplement: Supplementary file 2 [file Table_2.DOCX]

**Supplementary Table 2.** Major compounds identified from partially purified fraction (EA80: MET20) of *Syzygium cumini* by GC- MS analysis

| **S.No** | **Name of the compound** | **Retention Time (min)** | **Peak area (%)** |
| --- | --- | --- | --- |
| 1. | Phenol, 4,4'-(1-methylethylidene)bis[2-methyl] | 14.94 | 28.6 |
| 2. | (-)-Quinic acid | 21.46 | 14.81 |
| 3. | Isovaleric acid, nonyl ester | 21.30 | 8.78 |
| 4. | 4,5,7-trihydroxy-2-Octenoic acid | 9.55 | 6.77 |
| 5. | 2,5-anhydro-1,6-dideoxyhexo-3,4-diulose | 9.74 | 2.61 |
| 6. | α-d-galactopyranoside | 10.38 | 2.76 |
| 7. | 2,4-dinitrophenylether | 30.21 | 2.81 |
| 8. | 2-Methyl-5-(2,6,6-trimethyl-cyclohex-1-enyl)-pentane-2,3-diol | 30.644 | 1.86 |
| 9. | 3-(4-Isopropylphenyl)-2-Methyl-1-Propanol | 27.708 | 1.74 |
| 10. | 4-Oxo-.beta.-isodamascol | 29.605 | 1.69 |
| 11. | 16-Keto-tetrahydrosolasodine | 29.275 | 1.60 |
| 12. | 2-(1,3-benzoxazol-2-ylsulfanyl)-n-(4,6-dimethoxy-2-pyrimidinyl)acetamide | 30.450 | 1.54 |
| 13. | 4,6-Decadiyne | 14.693 | 1.44 |
| 14. | 2,5-Furandione, 3-(1,1-dimethylethyl) | 9.947 | 1.36 |
| 15. | (-)-delta.-Panasinsine | 30.317 | 1.12 |
| 16. | Ethyl(dimethyl)isopropoxysilane | 12.498 | 1.11 |
| 17. | 1-[5-hydroxy-4-methyl-2-hexenyl]cyclohexanol | 21.633 | 1.07 |
| 18. | 1,2-di-tert-butylbenzene | 28.448 | 1.02 |
| 19. | bis(Hex-5-en-1-yloxy)(dimethyl)silane | 23.142 | 1.00 |
