# Supplementary figures and images for: Synergistic Effect of Quinic Acid Derived From Syzygium cumini and Undecanoic Acid Against Candida spp. Biofilm and Virulence
Source: Front Microbiol. 2018 Nov 26;9:2835. doi: 10.3389/fmicb.2018.02835 (PMC6275436; doi:10.3389/fmicb.2018.02835)

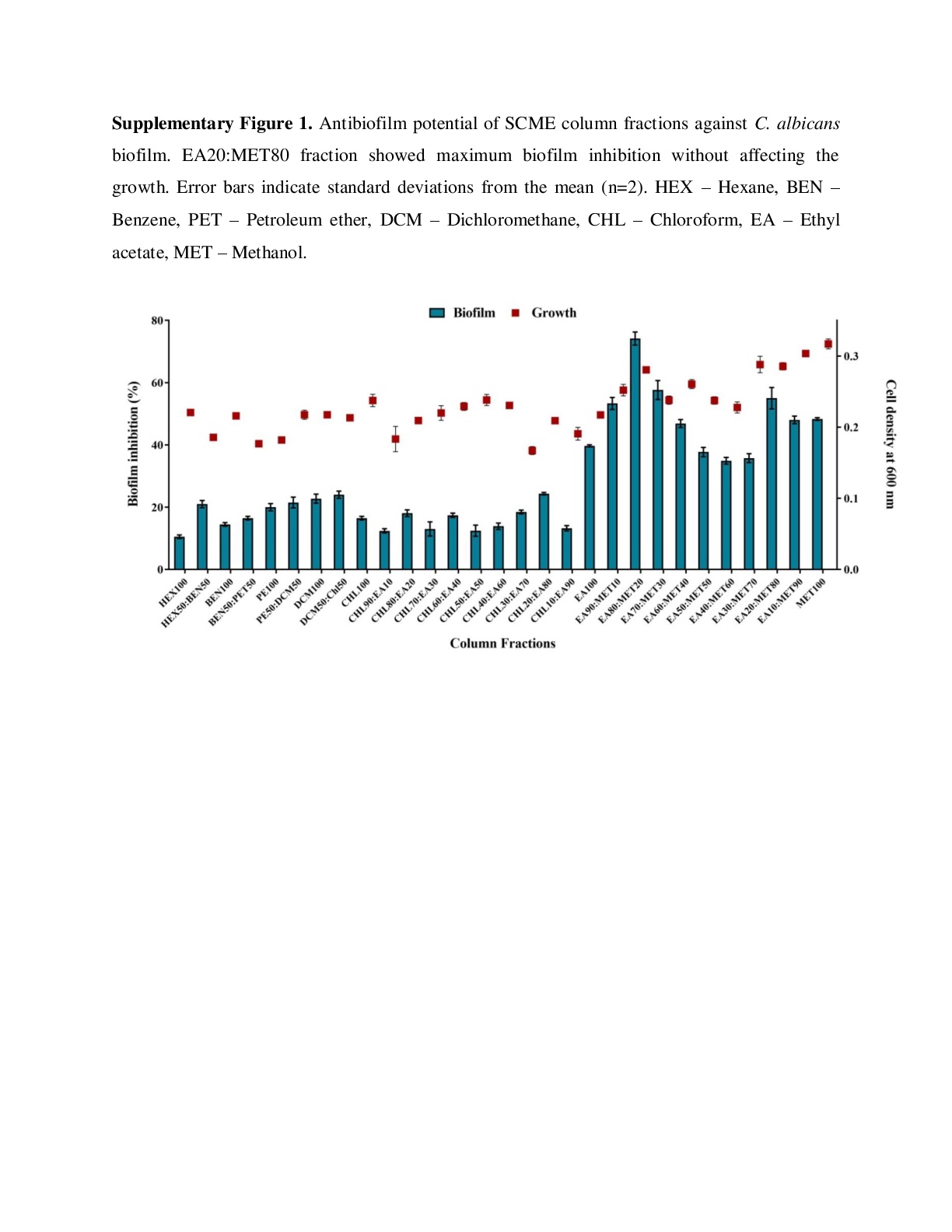

Supplement: Supplementary file 3 [file Image_1.JPEG]

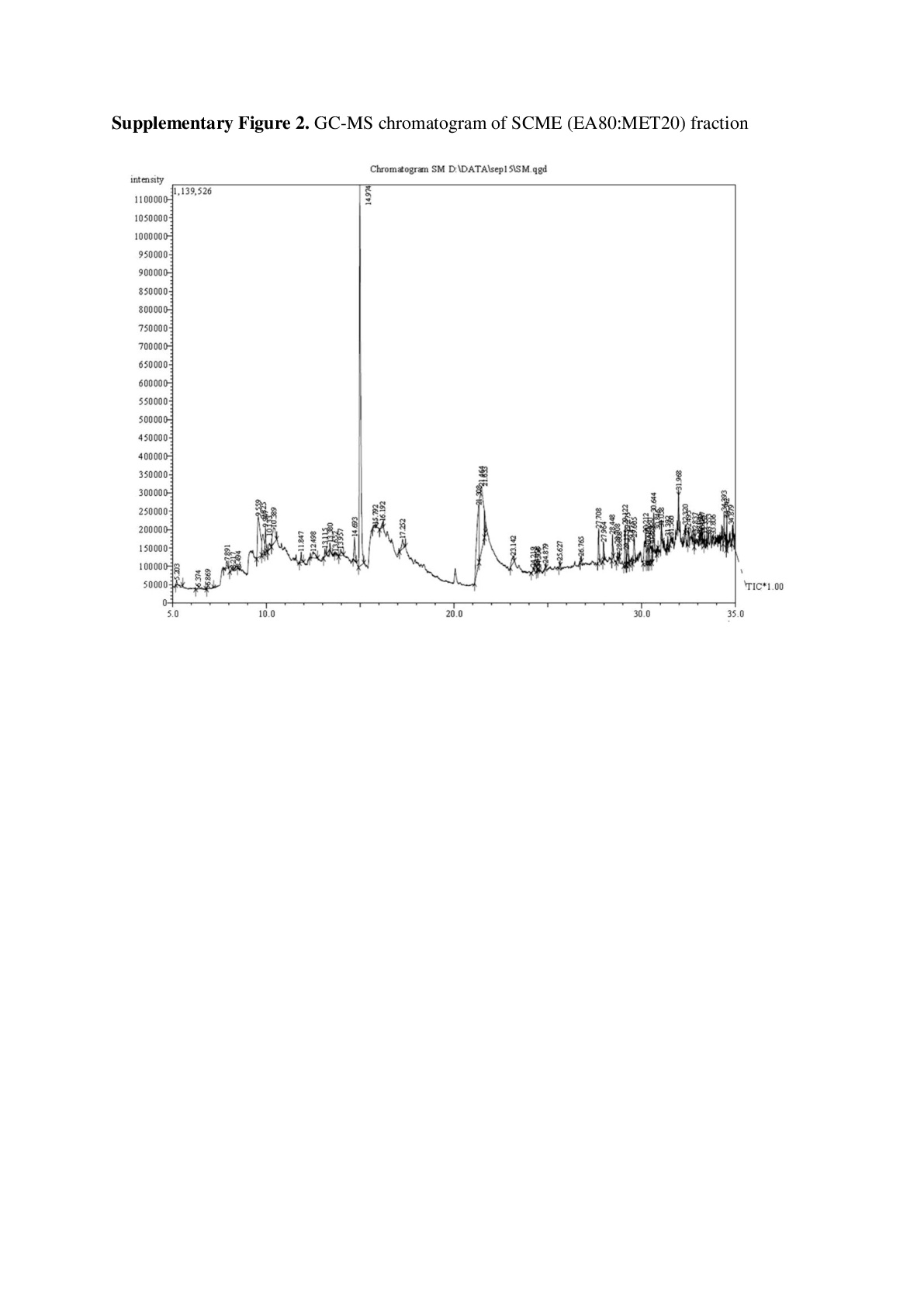

Supplement: Supplementary file 4 [file Image_2.JPEG]

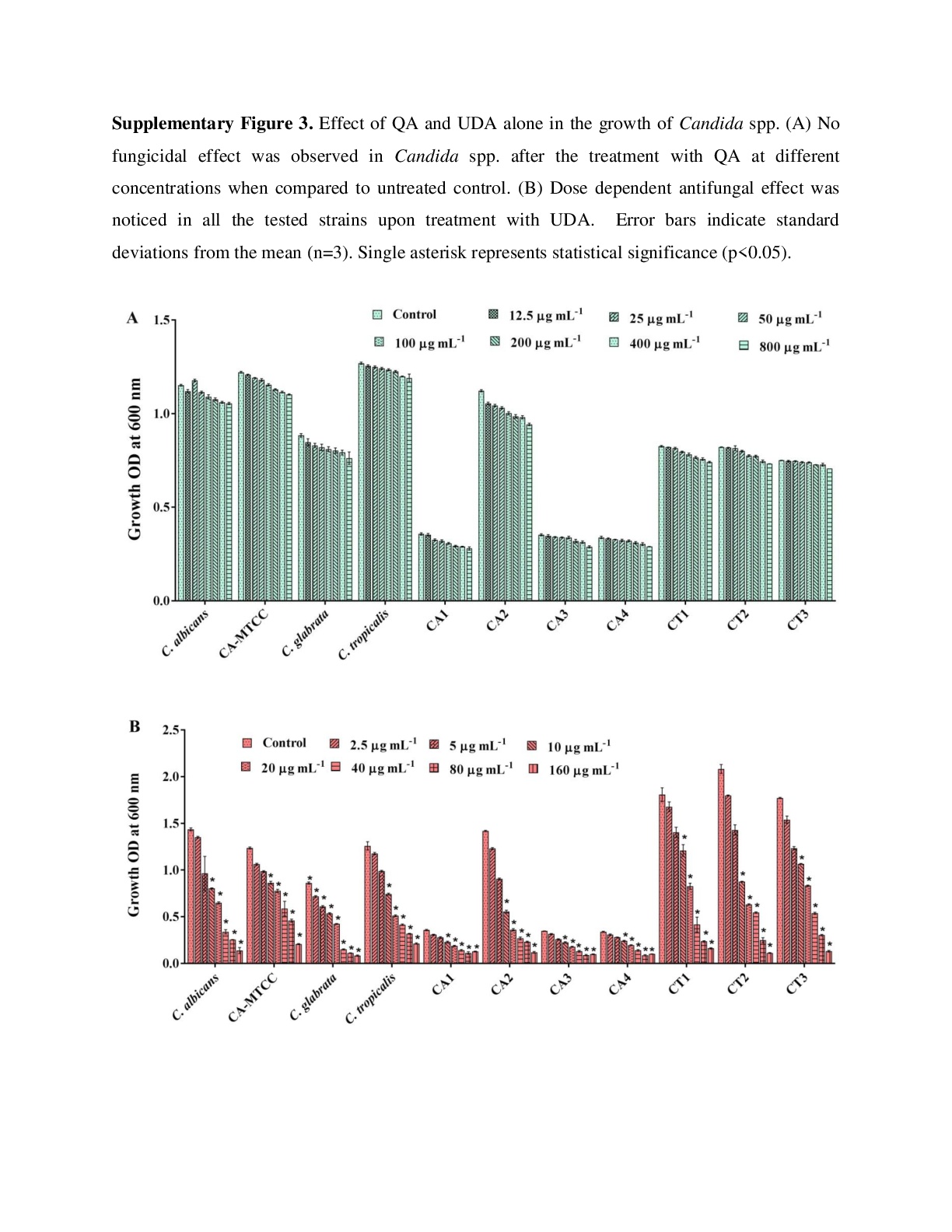

Supplement: Supplementary file 5 [file Image_3.jpg]
